# Supplementary material for: Modeling glioblastoma heterogeneity as a dynamic network of cell states
Source: Mol Syst Biol. 2021 Sep 16;17(9):e10105. doi: 10.15252/msb.202010105 (PMC8444284; doi:10.15252/msb.202010105)
Supplement: Supplementary file 6 — Source Data for Figure 5 [file MSB-17-e10105-s004.zip › Figure5A_sourcedata/GSEA_3071/nosigdb_stateV.GseaPreranked.1622795159954/GANDAL_IBD_UP_PMID29439242.html]

Details for gene set GANDAL\_IBD\_UP\_PMID29439242[GSEA]

|  || Dataset | state43071 |
| Phenotype | NoPhenotypeAvailable |
| Upregulated in class | na\_neg |
| GeneSet | GANDAL\_IBD\_UP\_PMID29439242 |
| Enrichment Score (ES) | -0.64449984 |
| Normalized Enrichment Score (NES) | -3.2962816 |
| Nominal p-value | 0.0 |
| FDR q-value | 0.0 |
| FWER p-Value | 0.0 |
Table: GSEA Results Summary

  

Fig 1: Enrichment plot: GANDAL\_IBD\_UP\_PMID29439242      
 Profile of the Running ES Score & Positions of GeneSet Members on the Rank Ordered List

  

| PROBE | GENE SYMBOL | GENE\_TITLE | RANK IN GENE LIST | RANK METRIC SCORE | RUNNING ES | CORE ENRICHMENT || 1 | PDPN |  |  | 65 | 1.493 | 0.0112 | No |
| 2 | TNC |  |  | 246 | 1.123 | -0.0054 | No |
| 3 | CXCR4 |  |  | 550 | 0.830 | -0.0502 | No |
| 4 | PCDH17 |  |  | 658 | 0.770 | -0.0584 | No |
| 5 | ST3GAL4 |  |  | 839 | 0.684 | -0.0820 | No |
| 6 | COL1A2 |  |  | 1028 | 0.614 | -0.1082 | No |
| 7 | LPCAT1 |  |  | 1281 | 0.537 | -0.1479 | No |
| 8 | RAB31 |  |  | 1604 | 0.459 | -0.2022 | No |
| 9 | PDE4B |  |  | 1660 | 0.444 | -0.2057 | No |
| 10 | EGR1 |  |  | 1691 | 0.438 | -0.2045 | No |
| 11 | C1R |  |  | 1696 | 0.437 | -0.1983 | No |
| 12 | IL7R |  |  | 1761 | 0.425 | -0.2038 | No |
| 13 | PLAU |  |  | 1863 | 0.408 | -0.2167 | No |
| 14 | ANXA1 |  |  | 2165 | 0.366 | -0.2684 | No |
| 15 | FOS |  |  | 2529 | 0.319 | -0.3327 | No |
| 16 | PRRX1 |  |  | 2674 | 0.302 | -0.3555 | No |
| 17 | CLDN1 |  |  | 2767 | 0.293 | -0.3684 | No |
| 18 | PRDM1 |  |  | 2921 | 0.277 | -0.3933 | No |
| 19 | LCP1 |  |  | 3470 | -0.294 | -0.4934 | No |
| 20 | IFITM2 |  |  | 3585 | -0.312 | -0.5102 | No |
| 21 | GJA1 |  |  | 3602 | -0.316 | -0.5083 | No |
| 22 | BHLHE40 |  |  | 3620 | -0.318 | -0.5065 | No |
| 23 | CASP1 |  |  | 3648 | -0.323 | -0.5065 | No |
| 24 | KLHL5 |  |  | 3762 | -0.342 | -0.5227 | No |
| 25 | LY96 |  |  | 3829 | -0.354 | -0.5297 | No |
| 26 | GZMB |  |  | 3906 | -0.368 | -0.5384 | No |
| 27 | ITGB2 |  |  | 3948 | -0.377 | -0.5403 | No |
| 28 | CMTM7 |  |  | 4056 | -0.400 | -0.5544 | No |
| 29 | HIF1A |  |  | 4130 | -0.417 | -0.5618 | No |
| 30 | TRIM22 |  |  | 4138 | -0.419 | -0.5565 | No |
| 31 | CYR61 |  |  | 4384 | -0.497 | -0.5954 | No |
| 32 | IFITM3 |  |  | 4424 | -0.509 | -0.5948 | No |
| 33 | SLC7A5 |  |  | 4513 | -0.547 | -0.6030 | No |
| 34 | IGFBP7 |  |  | 4622 | -0.604 | -0.6141 | No |
| 35 | SAMD9L |  |  | 4638 | -0.613 | -0.6072 | No |
| 36 | CD55 |  |  | 4834 | -0.742 | -0.6328 | Yes |
| 37 | THY1 |  |  | 4874 | -0.781 | -0.6278 | Yes |
| 38 | TGM2 |  |  | 4928 | -0.842 | -0.6246 | Yes |
| 39 | KDELR3 |  |  | 4974 | -0.893 | -0.6191 | Yes |
| 40 | IGFBP5 |  |  | 4978 | -0.897 | -0.6055 | Yes |
| 41 | ME1 |  |  | 4982 | -0.899 | -0.5918 | Yes |
| 42 | WARS |  |  | 5014 | -0.956 | -0.5826 | Yes |
| 43 | COL7A1 |  |  | 5041 | -1.021 | -0.5714 | Yes |
| 44 | GBP1 |  |  | 5051 | -1.031 | -0.5568 | Yes |
| 45 | KLF2 |  |  | 5083 | -1.110 | -0.5452 | Yes |
| 46 | GLIPR1 |  |  | 5109 | -1.177 | -0.5313 | Yes |
| 47 | TIMP1 |  |  | 5134 | -1.251 | -0.5161 | Yes |
| 48 | TGFBI |  |  | 5136 | -1.259 | -0.4963 | Yes |
| 49 | XBP1 |  |  | 5165 | -1.393 | -0.4796 | Yes |
| 50 | ALPL |  |  | 5166 | -1.396 | -0.4575 | Yes |
| 51 | FKBP11 |  |  | 5167 | -1.399 | -0.4354 | Yes |
| 52 | GPX8 |  |  | 5171 | -1.418 | -0.4135 | Yes |
| 53 | PLAUR |  |  | 5185 | -1.479 | -0.3926 | Yes |
| 54 | S100A11 |  |  | 5196 | -1.536 | -0.3702 | Yes |
| 55 | RARRES3 |  |  | 5206 | -1.582 | -0.3468 | Yes |
| 56 | BGN |  |  | 5216 | -1.654 | -0.3224 | Yes |
| 57 | DCN |  |  | 5233 | -1.798 | -0.2969 | Yes |
| 58 | CDH11 |  |  | 5245 | -1.943 | -0.2683 | Yes |
| 59 | MGP |  |  | 5252 | -2.037 | -0.2372 | Yes |
| 60 | COL4A1 |  |  | 5266 | -2.279 | -0.2036 | Yes |
| 61 | CTHRC1 |  |  | 5270 | -2.394 | -0.1663 | Yes |
| 62 | LUM |  |  | 5277 | -2.507 | -0.1277 | Yes |
| 63 | COL6A3 |  |  | 5293 | -3.722 | -0.0717 | Yes |
| 64 | KRT17 |  |  | 5295 | -4.538 | -0.0000 | Yes |
Table: GSEA details [plain text format]

  

Fig 2: GANDAL\_IBD\_UP\_PMID29439242: Random ES distribution      
 Gene set null distribution of ES for **GANDAL\_IBD\_UP\_PMID29439242**
